# Retinoic acid and arsenic trioxide induce lasting differentiation and demethylation of target genes in APL cells

**DOI:** 10.1038/s41598-019-45982-7

**Published:** 2019-07-01

**Authors:** Thomas T. Huynh, Mohammad Sultan, Dejan Vidovic, Cheryl A. Dean, Brianne M. Cruickshank, Kristen Lee, Chao-Yu Loung, Ryan W. Holloway, David W. Hoskin, David M. Waisman, Ian C. G. Weaver, Paola Marcato

**Affiliations:** 1 Department of Pathology, Dalhousie University, Halifax, NS Canada; 2 Department of Psychology and Neuroscience, Dalhousie University, Halifax, NS Canada; 3 Department of Microbiology and Immunology, Dalhousie University, Halifax, NS Canada; 4 Department of Biochemistry and Molecular Biology, Dalhousie University, Halifax, NS Canada; 5 Department of Psychiatry, Dalhousie University, Halifax, NS Canada; 6 Brain Repair Centre, Dalhousie University, Halifax, NS Canada

**Keywords:** Acute myeloid leukaemia, Experimental models of disease

## Abstract

Acute promyelocytic leukemia (APL) is characterized by arrested differentiation of promyelocytes. Patients treated with all-trans retinoic acid (ATRA) alone experience relapse, while patients treated with ATRA and arsenic trioxide (ATO) are often relapse-free. This suggests sustained changes have been elicited by the combination therapy. To understand the lasting effects of the combination therapy, we compared the effects of ATRA and ATO on NB4 and ATRA-resistant NB4-MR2 APL cells during treatment versus post treatment termination. After treatment termination, NB4 cells treated with ATRA or ATO reverted to non-differentiated cells, while combination-treated cells remained terminally differentiated. This effect was diminished in NB4-MR2 cells. This suggests combination treatment induced more permanent changes. Combination treatment induced higher expression of target genes (e.g., transglutaminase 2 and retinoic acid receptor beta), which in NB4 cells was sustained post treatment termination. To determine whether sustained epigenetic changes were responsible, we quantified the enrichment of histone modifications by chromatin immunoprecipitation, and CpG methylation by bisulfite-pyrosequencing. While ATRA and combination treatment induced similar histone acetylation enrichment, combination treatment induced greater demethylation of target genes, which was sustained. Therefore, sustained demethylation of target genes by ATRA and ATO combination treatment is associated with lasting differentiation and gene expression changes.

## Introduction

Acute promyelocytic leukemia (APL) accounts for approximately 10–15% of adult acute myeloid leukemias^1^ and until recently was associated with high mortality and poor patient outcomes^2^. The malignancy is characterized by arrested promyelocyte differentiation in the myeloid lineage of hematopoietic stem cells, resulting in the absence of mature granulocytes and an over accumulation of promyelocyte precursors^3^. The deficiency of this population of cells manifests in patients as severe coagulation defects that can culminate in fatal disseminated intravascular coagulation and systemic hemorrhaging^4^. Uniquely, the cause in 98% of APL cases is attributed to a single chromosomal translocation event between chromosome 15 and chromosome 17^5–7^. This results in aberrant fusion between the promyelocytic leukemia (*PML*) and retinoic acid receptor α (*RAR*α) genes located on the respective chromosomes. The resultant PML-RARα chimeric protein behaves as an altered RAR nuclear receptor, which changes the DNA binding specificity, and represses the transcriptional programs normally controlled by RAR-retinoid-X-receptor heterodimers, through enhanced interactions with corepressors^8–10^. Consequently, differentiation is abrogated and immortalization of the promyelocytes is promoted.

Similar to other malignancies, APL cells are characterized by aberrant epigenetic changes to DNA methylation and histone modifications which interfere with normal transcriptional programs and contribute to blocked granulocyte differentiation and disease progression^11^. Specifically, PML-RARα binding to target gene promoters (e.g., retinoic acid receptor beta, *RARβ*; transglutaminase 2, *TGM*2) is associated with decreased activating histone marks (e.g., acetylation of histone 3 lysine 9 and 14, H3K9/14ac), increased repressive histone marks (e.g., tri-methylation of histone 3 lysine, H3K9me3) and DNA hypermethylation, leading to repressed transcription and heterochromatin formation^12–16^. PML-RARα binding to DNA is sufficient to induce histone modification changes, while DNA methylation changes can occur independent of PML-RARα and are believed to be a late event in leukemogenesis and associated with loss of transcription factor binding^16^.

In the context of treatment, the oncogenic fusion gene provides an ideal target for therapeutic intervention, since supraphysiological levels of the RAR ligand, all-trans retinoic acid (ATRA), induces degradation of the PML-RARα in APL cells and restores normal RAR transcriptional programs^9,13,17^. ATRA-induced target genes such as *TGM2*, mediate the differentiation of leukemic promyelocytes into mature granulocytes, restoring normal coagulation dynamics^18,19^. In terms of its effects on the aberrant epigenome of APL cells, ATRA induces genome-wide activating histone acetylation (e.g., H3K9ac and H3K9/14ac) of target genes, but it has negligible effects on histone methylation (e.g., H3K9me3 and H3K27me3) and DNA methylation^12–16^. Since the first introduction of ATRA therapy for APL in 1985, it has had a dramatic impact in the survival outcomes of this once deadly disease^20^. Used alone, ATRA induces a short-term remission in approximately 80% of patients^21–24^. Its subsequent combination with anthracycline-based chemotherapies significantly reduced relapse and increased response rates. Recent trials have demonstrated that the combination of ATRA with arsenic trioxide (ATO) treatment is superior to the combination of ATRA and anthracyclines; as a result, treatment recommendations are moving towards this newer combination^22,25–30^.

ATO induces degradation of PML-RARα, modest differentiation and apoptosis of APL cells, and is synergistic with ATRA^22,31–33^. The underlying mechanism behind the reduced relapse rates of the combination treatment is only partly understood and the effects of the combination treatment on epigenetic modifications have not been explored. In this study, we compare the short-term, long-term and the post treatment termination effects of ATRA and ATO on NB4 and ATRA-resistant NB4-MR2 APL cells, and characterized the epigenetic modifications of canonical target and differentiation genes *RARβ* and *TGM2*. The enhanced effectiveness of ATRA and ATO combination treatment in inducing terminal differentiation and expression of target genes was most evident 96 h after treatment was terminated. This effect was significantly diminished in ATRA-resistant NB4-MR2 cells. ATRA and ATO combination treatment reduced CpG island methylation of target gene promoters, which was sustained even after treatment was terminated. Together, this data provides new evidence of the benefits of ATRA and ATO post treatment termination and possible underlying epigenetic mechanisms for the reduced relapse associated with the combination treatment.

## Methods

### Cell line and culture conditions

NB4 cells (obtained from DSMZ) and NB4-MR2 cells (kindly provided by Dr. Wilson Miller Jr., McGill University, Montreal, QC)^34^ and cultured in suspension in cell culture grade flasks at 37 °C in a humidified atmosphere of 5% CO2 using RPMI-1640 (Invitrogen, Thermo Fisher Scientific) supplemented with 10% fetal bovine serum (FBS) and antibiotic-antimycotic (Invitrogen, Thermo Fisher Scientific) solution.

### Treatment protocol

All-trans retinoic acid (ATRA, Sigma-Aldrich) was dissolved in DMSO as a 100 mM stock solution and arsenic trioxide (ATO, Sigma-Aldrich) was dissolved in NaOH as a 100 mM stock solution and stored in aliquots at −80 °C. Prior to use, they were serially diluted in media to working concentrations. NB4 or NB4-MR2 cells were seeded in RPMI-1640 medium into flasks under four conditions: no treatment, 1 µM ATRA, 0.5 µM or 1.5 µM ATO, and combination treatment (1 µM ATRA and 0.5 µM or 1.5 µM ATO) for 72 h. A portion of these cells were then collected for various assays at the 72 h timepoint. The remainder of the cells were then passaged, and the medium was refreshed with their respective treatment conditions to extend treatment for up to 168 h, and then cells were collected for various analyses. Alternatively, for the 96 h post treatment termination timepoint, the cells from their respective flask were collected by centrifugation at 72 h after treatment initiation, washed with phosphate buffered saline (PBS) to remove residual drug(s) and returned to culture with drug-free treatment medium for another 96 h, before termination of the experiment and collection of cells. To minimize overcrowding of the cells (which contributes to background cell death) in the no treatment, ATRA alone and low dose ATO conditions, a portion of the cells were removed at 72 h and more media was added at 120 h.

### Flow cytometry

Treated NB4 cells were collected at 3 timepoints for flow cytometry analysis (72 h, 168 h and, 96 h post treatment for 72 h). Cells were collected by centrifugation (500xg) and each sample was then washed in PBS and suspended in blocking buffer consisting of PBS supplemented with 1% FBS and 1% ethylenediaminetetraacetic acid (EDTA, Sigma-Aldrich) and incubated with 3 µL Alexafluor 488 conjugated anti-human CD11b monoclonal mouse antibody (clone M1/70.15, Invitrogen, Thermo Fisher Scientific) in 100 µL of PBS containing 1% FBS, 1% EDTA for 30 min at 4 °C. Afterwards, the cells were centrifuged at 500xg and resuspended in PBS containing 1% FBS, 1% EDTA and 7‐aminoactinomycin D (7‐ADD, Biolegend) diluted to 1/50. A fluorescence activated cell sorter (FACS) Calibur or Canto (BD Pharmingen) was then used to detect the percentage of differentiated cells (CD11b + only or CD11b + and 7-AAD+) and dead undifferentiated cells (7-AAD only). The flow cytometry data was analyzed using FCS Express 4 Research Edition software (De Novo Software).

### Quantitative PCR

Total RNA was extracted from treated NB4 cells at two timepoints (72 h and at 96 h post treatment termination) using Trizol (Invitrogen, Thermo Fisher Scientific) and a Purelink RNA purification kit (Invitrogen, Thermo Fisher Scientific), following the manufacturer’s instructions, with the addition of an on-column DNase I (Invitrogen) treatment step. Equal amounts of RNA (0.25 µg) was converted into cDNA using an iScript cDNA Synthesis Kit (Bio‐Rad) according to the manufacturer’s recommendations. Quantitative PCR (qPCR) was performed by using SsoFast EvaGreen Supermix (Bio‐Rad) and gene‐specific primers (Supplemental Table 1) with a CFX384 thermocycler Touch real‐time PCR detection system (Bio‐Rad). Standard curves were generated for each primer set, and primer efficiencies were incorporated into the CFX Manager software (Bio‐Rad). The mRNA levels of each sample was calculated relative to two reference genes (HRPT1 and TBP) and normalized to their respective no treatment controls.

### Bisulfite pyrosequencing

Using a Purelink Genomic DNA mini kit (Invitrogen Thermo Fisher Scientific) and following the manufacturer’s instructions, genomic DNA was extracted from treated NB4 cells at 72 h and 96 h post treatment termination timepoints. Based on previous published methods, gene promotor specific DNA‐methylation was analyzed by sodium bisulfite pyrosequencing on a PyroMark Q24 Advanced pyrosequencer using the DNA EpiTect Fast DNA Bisulfite Kit and PyroMark PCR Kit (Qiagen) according to the manufacturer’s instructions. Three custom assays covering the *LINE-1*, *RARβ* and *TGM2* promoters were designed using PyroMark Assay Design software (v2.0; Qiagen N.V, Venlo, The Netherlands) and validated to amplify single PCR products (LINE-1 = 400nt, *RARβ* = 400nt, *TGM2* = 428nt) and the primer sequences listed in Supplemental Table 2. PCR conditions for both assays: 95 °C, 15 minutes; (94 °C, 30 sec; 56 °C, 30 sec; 72 °C, 30 sec) × 50 cycles; and 72 °C, 10 min.

### Statistical analysis

All statistical analyses were performed with GraphPad Prism Version 7. ANOVA (one‐way analysis of variance or repeated measures) was performed followed by post‐tests Dunnett or Bonferroni (specified in the figure legends), when multiple comparisons were made. Significant p values are represented as follows: * < 0.05.

## Results

### ATRA and ATO combination treatment sustains differentiation and results in cell death of NB4 cells post treatment termination

**Figure 1:**
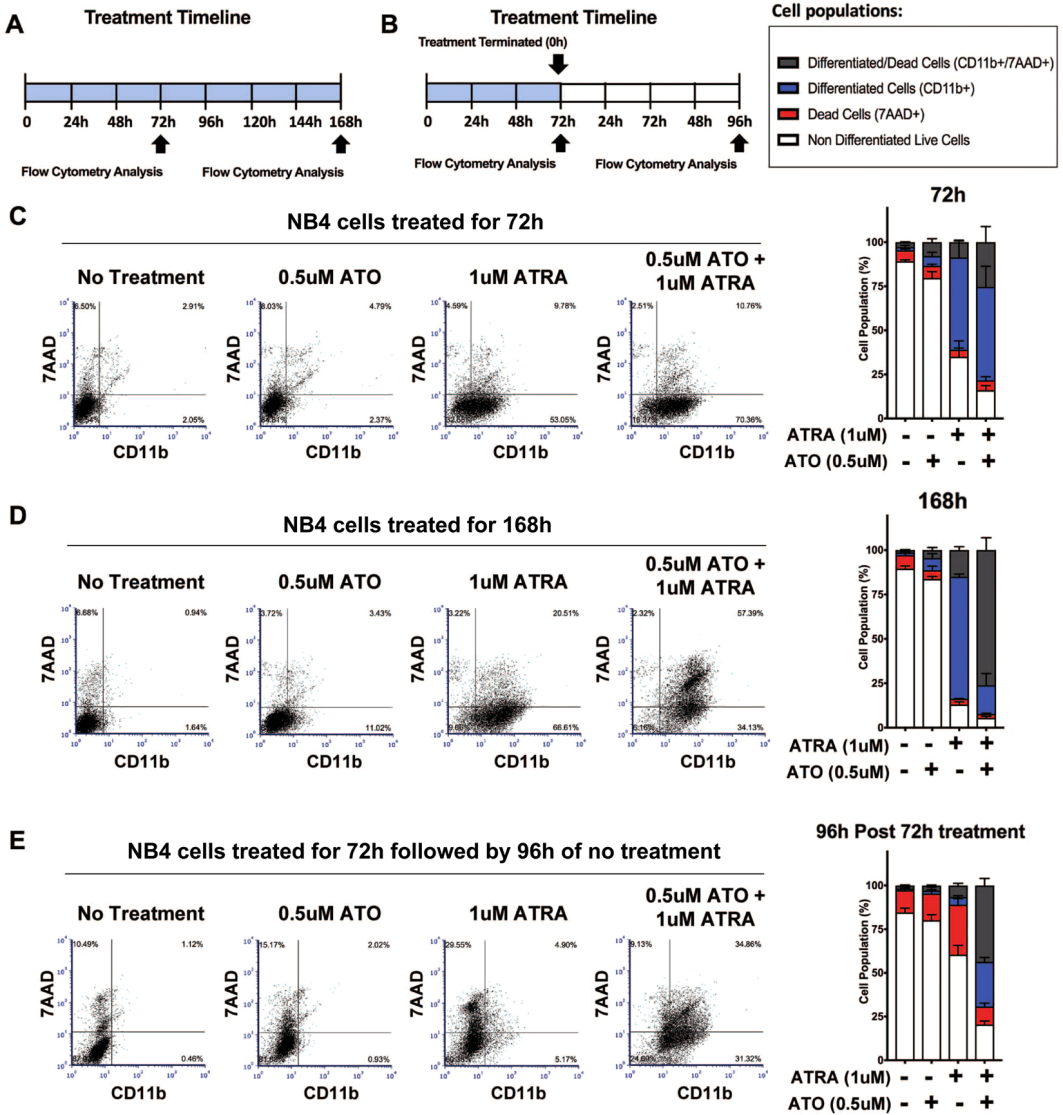
Combination 1 µM ATRA and 0.5 µM ATO treatment sustains differentiation and death of NB4 cells 96 h post treatment termination. (**A**) Schematic of treatment timeline and the timepoints NB4 cells samples were analyzed in (**C** and **D**). (**B**) Schematic of treatment timeline and the timepoint NB4 cells samples were analyzed in (**E**). (**C**–**E**) Representative flow cytometry dot plots of CD11b+, 7-AAD+, and CD11b+ /7-AAD+ NB4 cells under no treatment, 0.5 µM ATO, 1 µM ATRA, 0.5 µM ATO + 1 µM ATRA treatment after 72 h of continuous treatment (**C**), or 168 h of continuous treatment (**D**), or after 72 h treatment and subsequent 96 h post treatment termination. (**C**–**E**) The stacked bar graphs summarize the results of dot plots (n = 4, error bars represent standard deviation).

While ATRA alone induces short-term remission in APL patients^22^, the complete remission induced by daily dosage of 45 mg/m^2^ATRA and 0.15 mg/kg ATO combination treatment^25,27,28,35,36^, suggests the combination treatment is better at inducing long-term lasting effects, which are sustained post treatment termination. Pharmacokinetic studies in patients suggests that these doses translate to peak plasma concentrations of approximately 1 µM for ATRA^37,38^, while ATO has been reported as peaking at 6.85 µM before troughing to less than 1 µM^33^, to ranging from 0.08 to 0.40 µM^39^, or from 0.11 to 0.37 µM^40^. Therefore, to study the effect of these drugs alone or in combination for maintaining differentiation or inducing cell death post treatment termination, we treated the APL cell line NB4 or the MR2 ATRA-resistant clone with 1 µM ATRA and 0.5 µM ATO (Fig. 1) or 1.5 µM ATO (Supplemental Fig. 1), alone or in combination, to capture the range of potential ATO doses in patient circulation. We treated the cells continuously for 72 h or 168 h (Fig. 1A). Alternatively, after 72 h, treatment was terminated by washing the cells and subsequently culturing the cells for an additional 96 h in treatment-free medium (Fig. 1B). We determined the percentage of differentiated cells (surface expression of myeloid marker CD11b) and dead cells (7-AAD staining) under these various treatment conditions by flow cytometry. The combination of 1 µM ATRA and 0.5 µM ATO induced a larger population of differentiated CD11b positive cells in comparison to single ATRA and ATO treated cells after 72 h (Fig. 1C). As described previously, the higher dose of ATO induced greater cell death (Supplemental Fig. 1)^29,41–43^. The population of differentiated cells (and differentiated cells that had died) became significantly more pronounced at 168 h (CD11b/7-ADD positive cells, Fig. 1D). However, the potential synergistic benefits of ATRA and ATO combination treatments became most evident after treatment was terminated for 96 h (Fig. 1B,E, Supplemental Fig. 1). Ninety-six hours post treatment termination, most of the cells treated with the single agents now lacked staining for the differentiation and death markers (Fig. 1E). We noted that the background cell death had increased 96 h post treatment termination. This was possibly due to overcrowding of the proliferating cells or stress that was induced by washing the cells.

Most strikingly and in sharp contrast to cells treated with single agents, the combination 1 µM ATRA + 0.5 µM ATO treated cells were still mostly differentiated and/or dead at 96 h post treatment termination (CD11b/7-ADD positive cells, Fig. 1D). This data mimics the clinical findings^20,22,26,27^, whereby ATRA and ATO combination therapy result in sustained effects, which persist after the termination of therapy.

**Figure 2:**
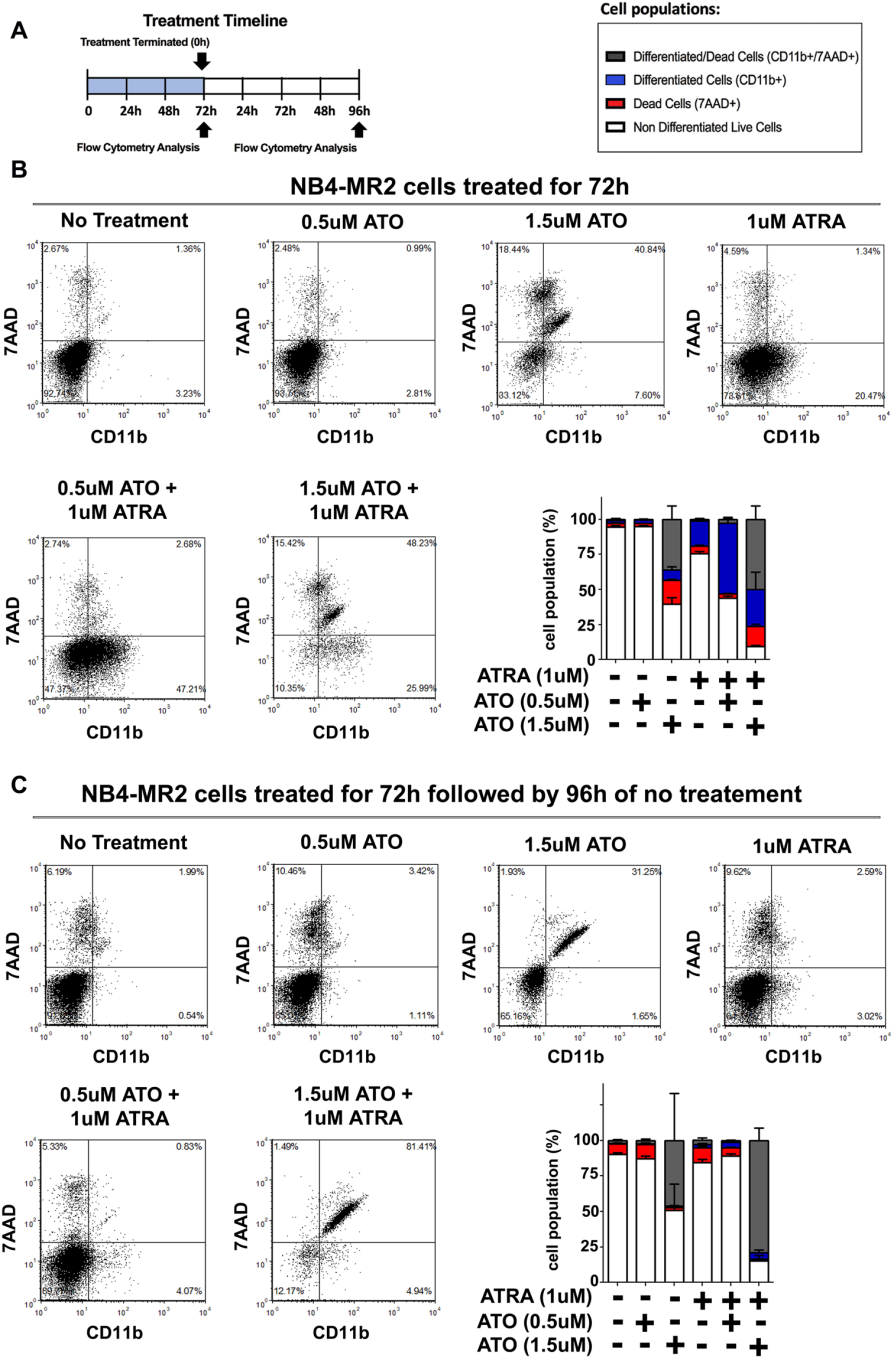
ATRA and ATO treatment have a reduced effect on inducing and sustaining differentiation and cell death in NB4-MR2 cells. (**A**) Schematic of treatment timeline and the timepoints NB4-MR2 cell samples were analyzed in (**B** and **C**). (**B** and **C**) Representative flow cytometry dot plots of CD11b+, 7-AAD+, and CD11b+/7-AAD+ NB4-MR2 cells under no treatment, 0.5uM ATO, 1.5 µM ATO, 1 µM ATRA, 0.5 µM ATO+ 1 µM ATRA, or 1.5 µM ATO+ 1 µM ATRA treatment after 72 h of continuous treatment (**B**) and after treatment has been terminated for 96 h (**C**). The stacked bar graphs summarize the results of dot plots (n = 4, error bars represent standard deviation).

We applied the same treatment regimen to the ATRA-resistant NB4-MR2 cells (Fig. 2A,B). As expected, ATRA alone did not induce differentiation of the cells; however, the cells were partly differentiated by the ATRA and 0.5 µM ATO combination treatment, and predominately killed by 1.5 µM ATO (Fig. 2C). This is consistent with previously published findings, where the ATRA-resistant NB4-MR2 cells remain sensitive to ATO treatment^41–43^.

### Combination treatment of ATRA and ATO is more effective at maintaining high transcript levels of TGM2, RARβ, CCL2 and ASB2 in NB4 cells post treatment termination

**Figure 3:**
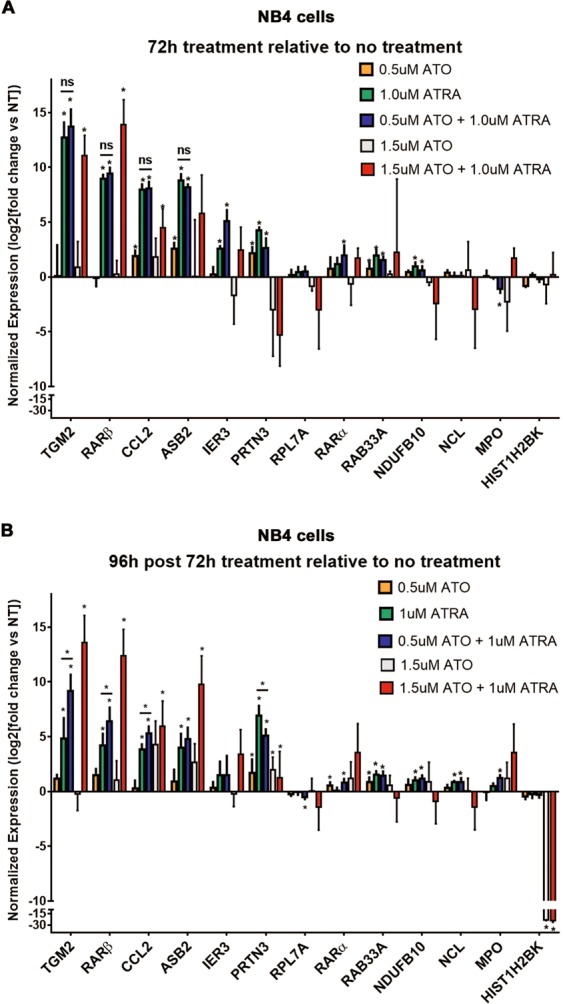
Combination treatment of ATRA and ATO is more effective at maintaining high levels of TGM2, RARβ, CCL2 and ASB2 mRNA 96 h post treatment termination. (**A** and **B**) QPCR analysis detects relative levels of mRNA of target genes in NB4 cells 72 h after treatment (**A**) and subsequent 96 h post treatment termination (**B**) with 0.5 µM ATO, 1.5 µM ATO, 1 µM ATRA, or combination treatments. The mRNA levels of target genes are log2 transformed and relative to the no treatment sample and reference genes (n = 4, error bars represent standard deviation, significance determined using one-way ANOVA with multiple comparisons, p value < 0.05 indicated by *). Notably, the 1.5 µM ATO, 1.5 µM ATO + 1 µM ATRA combination treatment samples were completed at a later time and compared to their own no treatment control.

Having observed that ATRA and ATO combination treatment maintain the majority of NB4 cells in a state of terminal differentiation after treatment had ended (Fig. 1E, Supplemental Fig. 1), we next wondered if gene expression changes were similarly more persistent. Gene expression changes are a key component of the interfered differentiation and symptoms of APL disease^44^. Supraphysiological levels of ATRA can restore the expression of epigenetically silenced target genes in APL cells^13^. Using QPCR, we determined the effect of ATRA and ATO individually or in combination at 72 h and following 96 h post treatment termination, on the mRNA levels of several genes involved in APL processes such as leukemic differentiation (TGM2, RARβ), granulocyte function (MPO, PRTN3) and other ATRA-regulated targets (e.g., CCL2) in comparison to single agent treatment^15,44^. ATRA alone and combination treatment resulted in significantly higher transcript levels of several genes (TGM2, RARβ, CCL2, ASB2, RPL7A, RARα, RAB33A, NDUFB10, NCL and HIST1H2BK) after 72 h of treatment (Fig. 3A). ATO treatment alone had a comparatively minor effect on expression of the genes. Notably, for most of the genes, there was no significant difference in the transcript levels induced by ATRA alone and combination treated cells (Fig. 3A). One notable exception was RARβ, which was induced to higher levels when NB4 cells were treated with combination treatment ATRA with higher dose 1.5 µM ATO. Interestingly, the sustained effects of the combination treatments over ATRA alone became much more apparent 96 h after termination of treatment, where the higher transcript levels of TGM2, RARβ, CCL2 and ASB2 were still present (Fig. 3B). Therefore, the combination treatments maintained greater transcript levels of these genes once treatment had been terminated. The sustained expression of certain target genes may explain why terminal granulocytic differentiation is sustained in combination treated cells in comparison to single agent treated cells, especially considering the key role that TGM2 has in differentiation of NB4 cells^18^.

### Combination treatment of ATRA and ATO fails to maintain high transcript levels of target genes in NB4-MR2 cells post treatment termination

**Figure 4:**
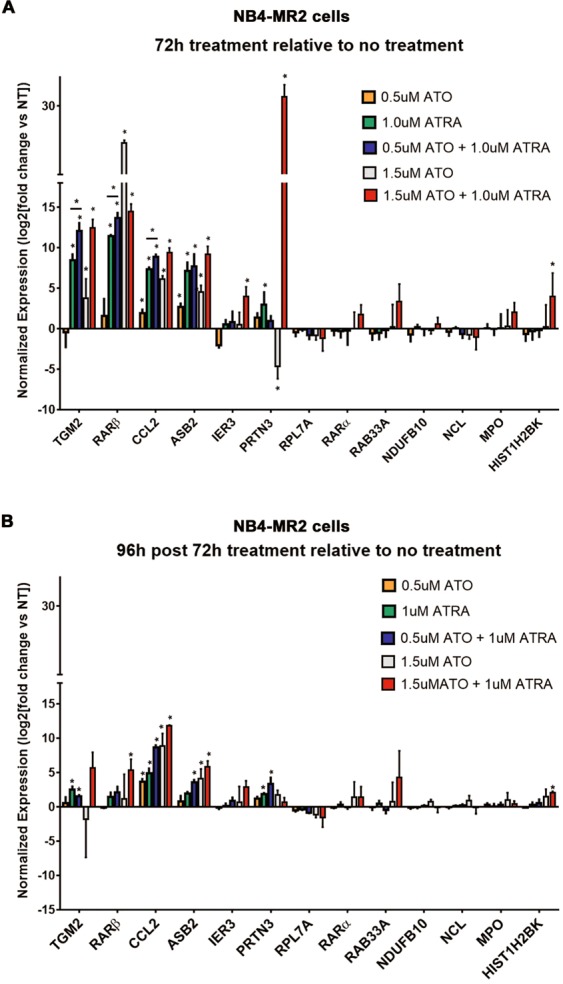
ATRA and ATO induce gene expression changes in NB4-MR2 cells, which are weakly sustained post treatment termination. (**A** and **B**) QPCR analysis detects relative levels of mRNA of target genes in NB4-MR2 cells 72 h after treatment (**A**) and subsequent 96 h post treatment termination (**B**) with 0.5 µM ATO, 1.5 µM ATO, 1 µM ATRA, or combination treatments. The mRNA levels of target genes are log2 transformed and relative to the no treatment sample and reference genes (n = 4, error bars represent standard deviation, significance determined using one-way ANOVA with multiple comparisons, p value < 0.05 indicated by*).

We compared the expression of these genes in NB4-MR2 cells under the same treatment conditions. Notably the ATRA-resistant clone has demonstrated altered ligand binding of PML/RAR-alpha and retinoid-induced gene expression^34^. We also found a somewhat different gene expression profile induced by the treatments at 72 h in NB4-MR2 cells, with generally more pronounced gene expression changes induced in the combination treatments (Fig. 4A). A notable exception was that much greater levels of RARβ was induced in the cells treated with higher dose 1.5 µM ATO alone (Fig. 4A). However, once treatment was terminated for 96 h, the gene expression changes that had been induced in the NB4-MR2 cells were only weakly sustained by the combination treatment ATRA with higher dose ATO (Fig. 4B). This is in sharp contrast to the NB4 cells, in which the combination treatment ATRA with even the lower dose ATO resulted in generally well sustained expression of genes (e.g. TGM2 and RARβ) at 96 h post treatment termination (Fig. 3B).

### ATRA induces sustained enrichment of H3K9/14ac at the *TGM2* and *RARβ* promoters in NB4 cells, which is not augmented by combination treatment

Decreased H3K9/14ac at target genes is key in their decreased expression in APL cells and ATRA-induced gene expression is associated with H3K9/14ac enrichment^15^. We therefore wondered if the greater sustained expression post treatment termination of some target genes induced by combination treatment (Fig. 3B) was due to greater enrichment of H3K9/14ac. Since *TGM2* and *RARβ* were most induced, with the greatest sustained expression by the combination treatment once treatment was terminated, we focused on these two genes for H3K9/14ac analysis by ChIP-qPCR. In agreement with previous reports, ATRA induced H3K9/14ac enrichment at *TGM2* and *RARβ* promoters (Supplemental Fig. 2A). The combination treatment of ATRA + 0.5 µM ATO did not further augment the H3K9/14ac enrichment at both 72 h and after 96 h post treatment termination. The lack of significant difference between the combination treatment versus the ATRA treatment alone (Supplemental Fig. 2A) suggests that H3K9/14ac enrichment may not play a critical role in the greater sustained expression of the genes induced by combination treatment (Fig. 3B).

We next wondered if perhaps the combination treatment decreases repressive H3K9me3 and H3K27me3 marks associated with silencing of *TGM2* and *RARβ* in APL cells^13^. Consistent with previous reports, ATRA had a minimal effect on H3K9me3 and H3K27me3 enrichment at the target genes (Supplemental Fig. 2B and C). The combination treatment also had minimal effects on the enrichment of the two repressive histone marks (Supplemental Fig. 2B and C). Overall, this indicates that the greater sustained TGM2 and RARβ mRNA levels induced by combination treatment (Fig. 3B) is probably not due to changes in these histone modifications (Supplemental Fig. 2).

### Combination treatment demethylates the CpG sites in the promoter regions of *TGM2* and *RARβ* in NB4 cells, and demethylation is sustained post t.reatment termination

**Figure 5:**
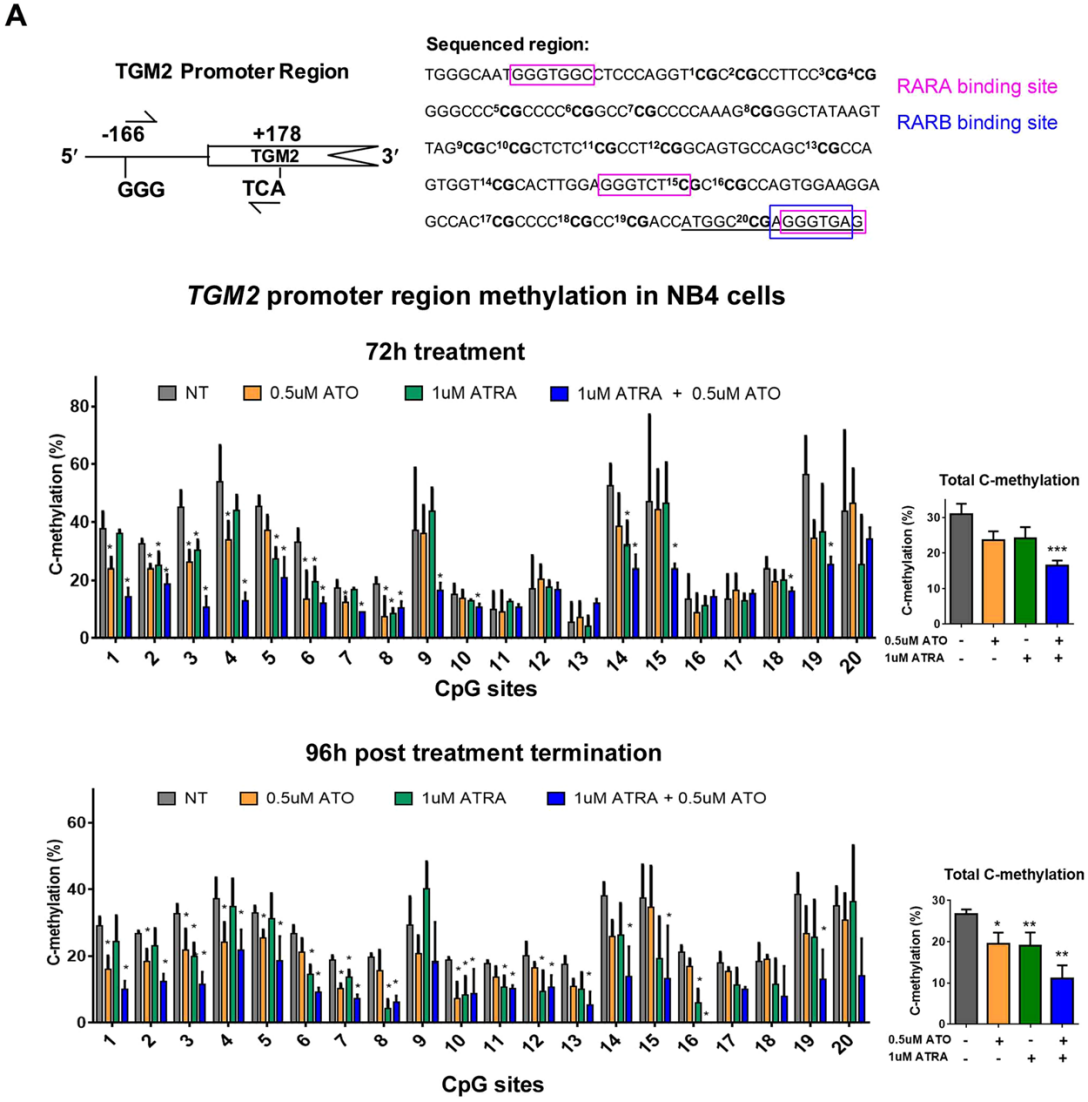
Combination 1 µM ATRA and 0.5 µM ATO treatment induces sustained demethylation of the CpG island in the promoter region of *TGM2* in NB4 cells. (**A**) Schematic representation of the *TGM2* promoter region and the specific 20 CpG sites located within the region that was bisulfite pyrosequenced. The binding sites for RARα and RARβ are indicated. (**B** and **C**) The methylation percentage of the individual 20 CpG sites and total C-methylation percentage of the region in NB4 cells following 72 h of treatment (**B**) and subsequent 96 h post treatment termination (**C**). Error bars represent standard deviation, significance determined using one-way ANOVA with multiple comparisons, n = 5.

CpG island DNA hypermethylation silencing of genes contributes to the arrested differentiation of granulocytes in APL^16^. Although ATRA is capable of inducing APL cells into a differentiated granulocytic phenotype, most evidence suggest that target genes, including differentiation-inducing gene *TGM2* and canonical target gene *RARβ*, remain aberrantly hypermethylated upon ATRA treatment^14,45^. However, we wondered if the combination treatment (or ATO) could be affecting the promoter methylation of the genes, contributing to the greater sustained transcript levels of the genes post treatment termination. Bisulfite pyrosequencing was used to interrogate the 20 CpGs in the CpG island in the *TGM2* promoter (Fig. 5A). Combination treatment of ATRA with 0.5 µM ATO significantly reduced the total C-methylation of the *TGM2* promoter region after 72 h treatment (Fig. 5B), and the demethylation was sustained 96 h after treatment had been terminated (Fig. 5C). Interestingly, the single agents did have some effect on methylation at individual sites and at 96 h after treatment had been terminated, ATRA and ATO alone did reduce overall methylation as well, albeit to lesser degree than the combination treatment. Notably, similar to the greater gene expression changes induced by combination ATRA treatment with the higher dose 1.5 µM ATO (Fig. 3), this combination also induced greater demethylation of the gene region (Supplemental Fig. 3). We noted that RARα and RARβ binding sites are in the *TGM2* promoter region (Fig. 5A), and CpG site 15 is located within a RARα binding site. CpG site 15 was among the more hypermethylated in the *TGM2* promoter region, and therefore its demethylation by the combination treatments (Fig. 5 and Supplemental Fig. 3), may be particularly important for expression of TGM2 (Fig. 3).

**Figure 6:**
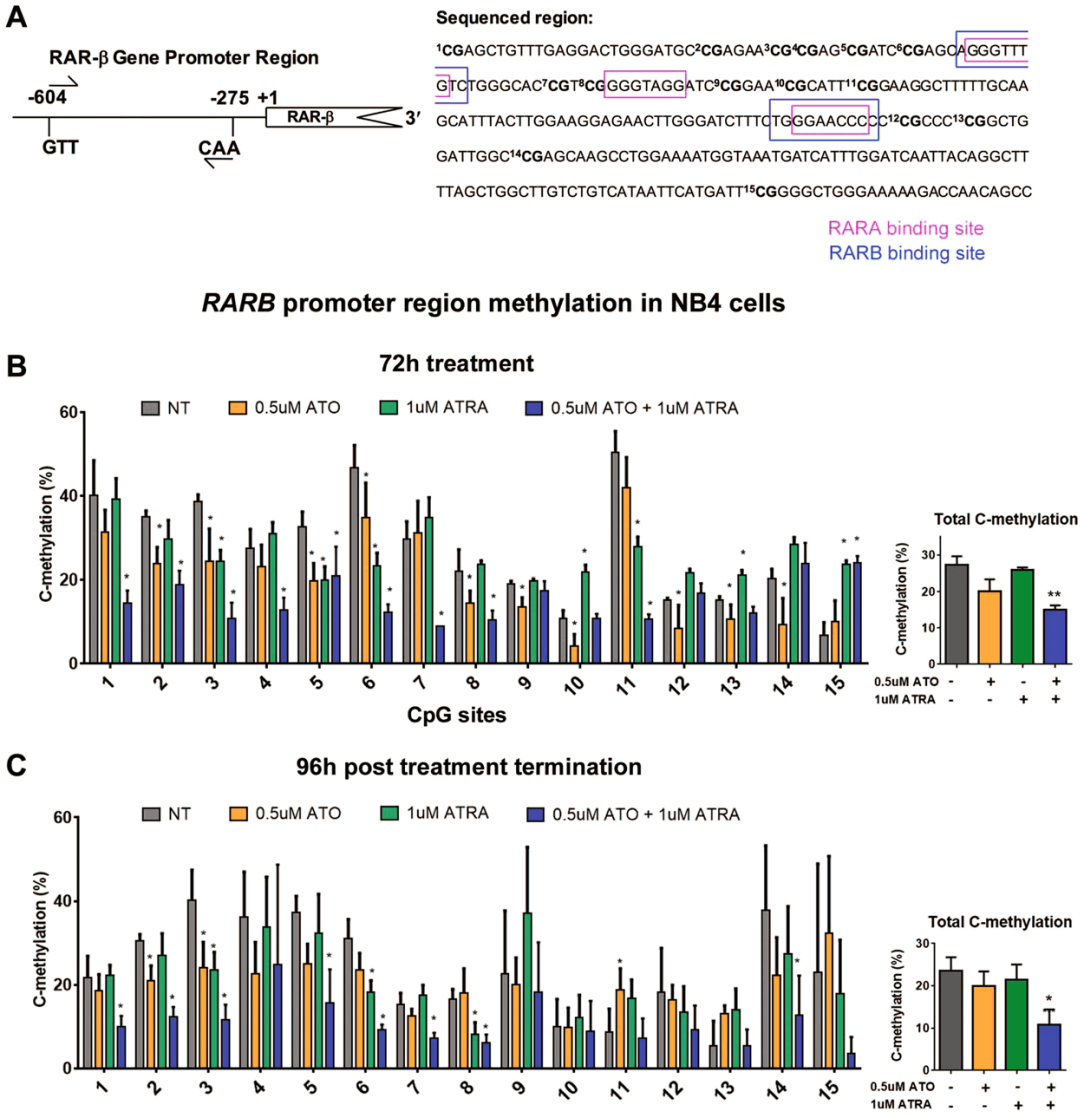
Combination 1 µM ATRA and 0.5 µM ATO treatment induces sustained demethylation of the CpG island in the promoter region of *RARβ* in NB4 cells. (**A**) Schematic representation of *RARβ* and the specific 15 CpG sites located within the region that were bisulfite pyrosequenced. The binding sites for RARα and RARβ are indicated. (**B** and **C**) The methylation percentage of the individual 15 CpG sites and total C-methylation percentage of the region in NB4 cells following 72 h of treatment (**B**) and subsequent 96 h post treatment termination (**C**). Error bars represent standard deviation, significance determined using one-way ANOVA with multiple comparisons, n = 5.

We also interrogated the methylation of the 15 CpG sites in the CpG island neighboring the *RARβ* transcription start that contains RARα and RARβ binding sites by bisulfite pyrosequencing (Fig. 6A). Again, treatment with ATRA or 0.5 µM ATO alone did not significantly alter the overall CpG methylation of the region, but combination treatment significantly reduced the total C-methylation of the *RARβ* promoter region at 72 h (Fig. 6B). Importantly, this effect was sustained 96 h after treatment termination (Fig. 6C). These demethylation effects were augmented in the combination ATRA and higher dose 1.5 µM ATO treated cells (Supplemental Fig. 4). Of note as well is the overall shift in CpG methylation at some sites when comparing 72 h versus 96 h post treatment conditions, even in the no treatment condition samples (e.g. sites 11 and 14, Fig. 6). This is possibly reflective of the dynamic methylation status of some of the CpG sites in the region that may be susceptible to changes in cell culturing conditions (e.g. cell crowding, spent media, increased background cell death). Regardless, there is still a significant and sustained decrease in methylation when the cells are treated with the combination treatments. Together, the *TGM2* and *RARβ* promoter analyses provides new evidence showing that the combination of ATRA and ATO reduces the aberrant methylation of key target genes, and similar to the transcript level analyses (Fig. 3), this effect was sustained after treatment had been terminated (Figs 5 and 6, and Supplemental Figs 3 and 4).

### Combination treatment demethylates of the CpG sites in the promoter regions of *TGM2* and *RARβ* in NB4-MR2 cells, but demethylation is not sustained post treatment termination

**Figure 7:**
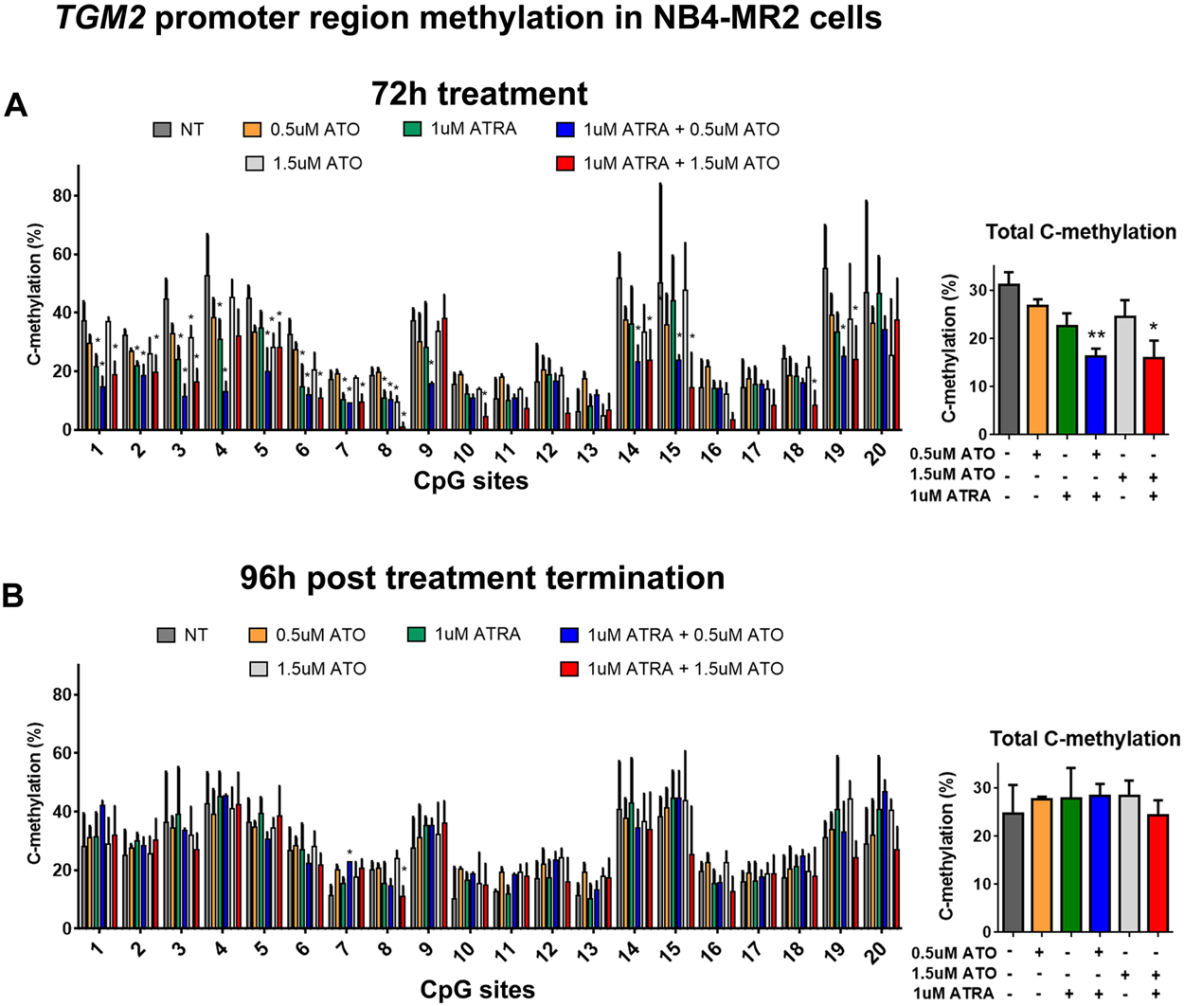
ATRA and ATO combination treatment demethylate the CpG island in the promoter region of *TGM2* in NB4-MR2 cells, but this is not sustained post treatment termination. (**A** and **B**) The methylation percentage of the individual 20 CpG sites and total C-methylation percentage of the region in NB4-MR2 cells following 72 h of treatment (**A**, n = 4) and subsequent 96 h post treatment termination (**B**, n = 3). Error bars represent standard deviation, significance determined using one-way ANOVA with multiple comparisons.

**Figure 8:**
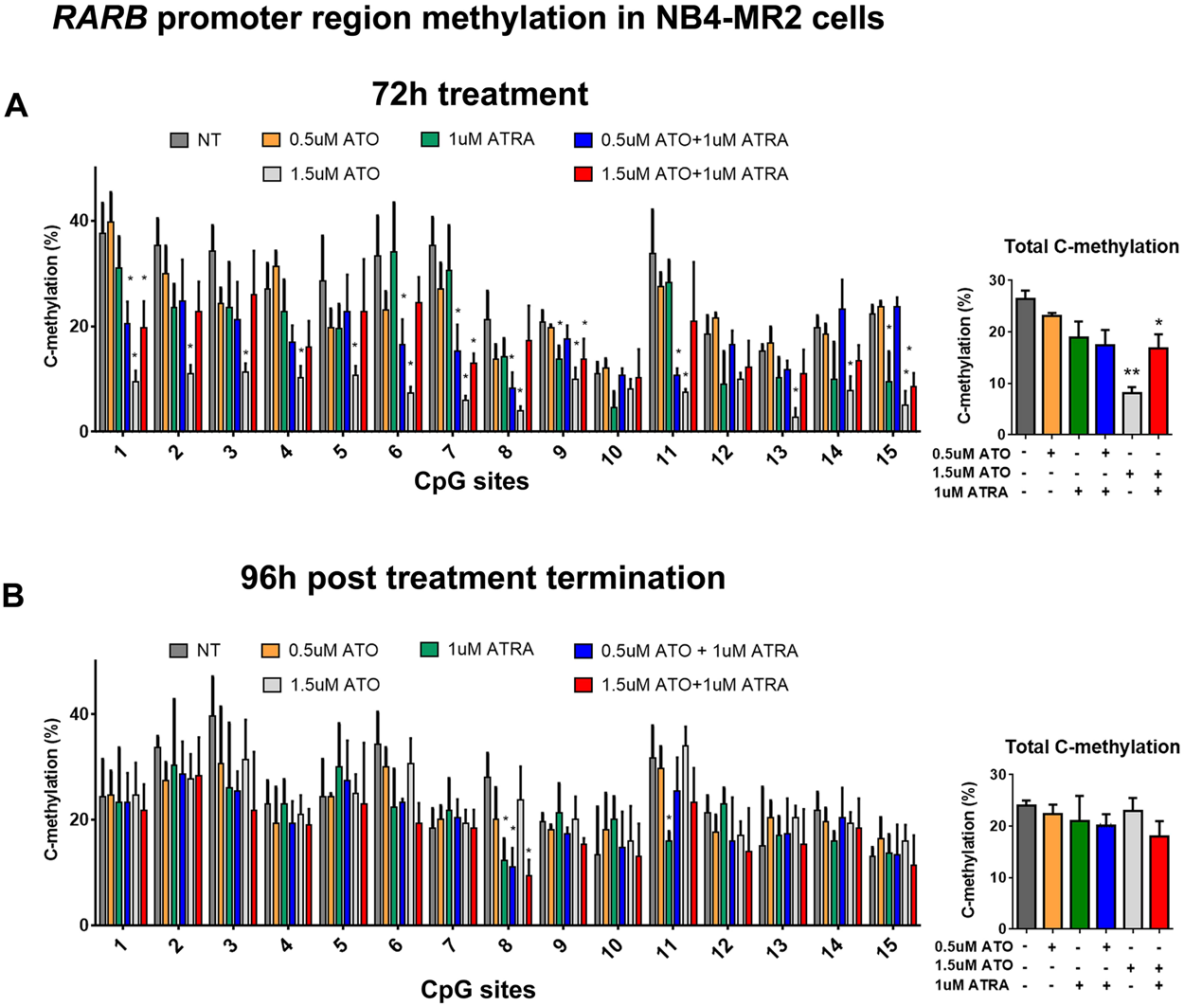
1.5 µM ATO demethylates the CpG island in the promoter region of *RARβ* in NB4-MR2 cells, but this is not sustained post treatment termination. (**A** and **B**) The methylation percentage of the individual 15 CpG sites and total C-methylation percentage of the region in NB4-MR2 cells following 72 h of treatment (**A**, n = 4) and subsequent 96 h post treatment termination (**B**, n = 3). Error bars represent standard deviation, significance determined using one-way ANOVA with multiple comparisons.

We next wondered how these treatment conditions would affect methylation of promoter regions of *TGM2* and *RARβ* in NB4-MR2 cells, in which gene expression changes were induced, but unlike NB4 cells, the gene expression changes were not sustained post treatment termination (Fig. 4). Bisulfite pyrosequencing revealed that the combination treatments demethylated the promoter region of *TGM2* in NB4-MR2 cells (Fig. 7A), but this effect was not sustained post treatment termination (Fig. 7B). These results mirrored the gene expression data for TGM2 in NB4-MR2 cells, which was also not sustained post treatment termination (Fig. 4). Intriguingly, for *RARβ,* higher dose 1.5 µM ATO treatment alone induced the greatest demethylation of the promoter region (Fig. 8A), which was again reflected in the gene expression changes (Fig. 4A). Importantly, these changes in *RARβ* methylation (like the changes in gene expression), were not sustained post treatment termination in NB4-MR2 cells (Fig. 4B). Together, this data strongly connects demethylation of the promoter regions of these genes with their increased expression.

### Global DNA methylation levels, represented by bisulfite pyrosequencing of *LINE-1*, are unchanged in ATRA, ATO or combination treated NB4 and NB4-MR2 cells

We next wondered if the effect of combined ATRA and ATO treatment on CpG methylation extended beyond the target genes and was genome-wide. Long interspersed nucleotide element 1 (*LINE-1*) elements are transposable repetitive elements making up 17% of genomic DNA and are often heavily methylated, holding up to a third of the methylation in the genome. As such, assessing methylation of *LINE-1* elements is an accepted surrogate for general global methylation levels of the genome^46–48^. Although this technique assesses relatively few CpG sites, *LINE-1* bisulfite pyrosquencing has been shown to reflect global DNA methylation changes with high significance^49^. We performed bisulfite pyrosequencing to assess methylation levels of 27 CpG sites within the promoter region of *LINE-1* subfamily *L1PA2*, which also lacks RARα and RARβ binding sites (Supplemental Fig. 5A)^46,50^. Overall, *LINE-1* methylation was unchanged by any of the treatment conditions at 72 h or 96 h after treatment was terminated in both NB4 cells (Supplemental Figs 5 and 6) and NB4-MR2 cells (Supplemental Fig. 7). This suggests that the reduced CpG methylation in response to treatment is associated with target genes (Figs 5–8), and is probably not global (Supplemental Figs 5–7).

## Discussion

Clinically, combined ATRA and ATO therapy is curative in APL patients, inducing long-lasting remission, whereas ATRA treatment alone is effective at eliciting short-term remission^22,25–28,51^. Previous studies of cultured APL cells describe the enhanced differentiation/cell death induction of the combination treatment over ATRA alone; however, these studies did not extend the analyses post treatment termination^32,52,53^. This study compares, for the first time, the effects of these treatments four days after treatment termination. Unexpectedly, we observed that under these conditions the effects of the combination treatment were much more dramatic. ATRA induced granulocytic differentiation in the short-term (72 h) and was increased further after long-term continuous treatment (168 h); however, ATRA-induced effects were largely lost once treatment had been terminated (Fig. 1). This is in sharp contrast to the combination treatment, which resulted in the differentiation and/or death of most the of the NB4 cells. Our analyses also revealed the greater overall efficacy achieved by treating APL cells with higher doses of ATO in combination with ATRA; the benefits of which become most apparent in the ATRA-resistant variant clone NB4-MR2 cells (Fig. 2). These findings model the long-lasting effects induced by the combination therapy in patients, which ATRA treatment alone comparably fails to do. The results further illustrate the potential benefit of higher dose ATO in combination with ATRA. The data from most clinical studies illustrating the benefit of ATRA and ATO combination treatment employ 0.15 mg/kg/day ATO^25,27,28,35,36^. Pharmacokinetic studies report a range of plasma ATO levels based on this dose^33,39,40^, although the 0.5 µM dose used in this study likely captures the lower reported plasma levels and the 1.5 µM may better represent the higher reported plasma levels. The results of a recent clinical trial utilizing 0.3 mg/kg/day ATO in combination with ATRA reported high rates of complete remission and overall survival^54^. From the current available patient data, it is unclear if ATRA administered with 0.3 mg/kg/day ATO versus 0.15 mg/kg/day ATO results in significantly different outcomes for APL patients.

In our analyses, the sustained effects of the ATRA and ATO combination treatment post treatment termination was also detected at the transcript level of some target genes in NB4 cells (Fig. 3B) and encouraged us to evaluate the epigenetic modifications of these genes. Epigenetic modifications such as histone marks and DNA methylation regulate gene transcription and are aberrant in APL resulting in silencing of many target genes^16,45^. Although the level of global methylation in APL NB4 cells remained unchanged by the combination treatments (Supplemental Figs 5 and 6), the methylation of target genes was observed (Figs 5 and 6). An important contributing factor to the increased effectiveness of combination treatment may be the sustained demethylation of target genes that are aberrantly methylated in APL cells (Figs 5 and 6). The aberrant methylation and silencing of *RARβ* is particularly well described in APL^14,15,45,55^. With the exception of one early study that used methylation-specific PCR to quantify *RARβ* methylation post ATRA treatment^55^, other later studies using more quantitative techniques report that ATRA treatment alone fails to revert the aberrant methylation of *RARβ* and other genes, including *TGM2*^14,15,45^. Using the quantitative bisulfite pyrosequencing technique, our results show that ATRA alone does have some effects on the methylation of individual CpG sites of the target genes; however, the combination treatments are much more effective at demethylating the CpG islands of *TGM2* and *RARβ* promoters (Figs 5–6)^14,15,45^.

With respect to ATO alone, there are reports of its effects on DNA methylation in APL cells. A recent study showed that 2.0 µM ATO reduced DNA methylation and increased mRNA levels of cell cycle-related genes in NB4 cells^56^. ATO reduced transcript levels of DNA methyltransferases 1, 3 A and 3B in NB4 cells, which should have genome-wide demethylating effects on DNA^56^. This is consistent with another study on the cell line HL-60 (an APL-like cell line that lacks the PML-RARα fusion), in which 1 µM ATO modestly reduced global methylation^57^. This suggests that more so than ATRA^14,15,45^, 1-2 µM ATO has demethylating effects in APL cells^56,57^. In our study ATO did not reduce global methylation as measured by *LINE-1* methylation, but the higher dose of 1.5 µM ATO did demethylate CpG sites in both NB4 and NB4-MR2 cells. Notably, in ATRA-resistant NB4-MR2 cells, 1.5 µM ATO alone induced the greatest demethylation of the CpG island in the *RARβ* promoter (Fig. 8A) and RARβ expression (Fig. 4A). However, these effects were not sustained post treatment termination (Figs 8B and 4B). Therefore, in general, our data is in agreement with those studies that concentrations of 1–2 µM ATO has at least some demethylating effects^56,57^. In future studies, it would be interesting to determine if prolonged continuous treatment of ATRA/ATO (i.e. 168 h of treatment), or if the drugs are administered consecutively instead of in combination, affects differentiation/cell death and methylation of target genes.

Therefore, while ATRA and ATO do appear to have some effects on DNA methylation, it is their combination that induces sustained demethylation of key target genes *TGM2* and *RARβ* post treatment termination (Figs. 5 and 6). Restoration of TGM2 levels in NB4 cells is necessary in ATRA-induced granulocyte differentiation^18^. The sustained reversal of aberrant methylation of the *TGM2* promoter (Fig. 5), and increased transcript levels (Fig. 3B) induced by the combination, could be a key event the sustained differentiation of NB4 cells post treatment termination (Fig. 1E). Notably, in the ATRA-resistant NB4-MR2 cells, TGM2 levels were increased and the gene demethylated by the combination, but these effects were not sustained post treatment termination. Therefore, the lack of sustained effects on DNA methylation could contribute to the resistance of NB4-MR2 cells to treatment.

The results from our gene-specific QPCR and bisulfite pyrosequencing studies highlight the need to perform transcriptome and genome-wide methylation analyses (e.g. whole genome bisulfite sequencing, or EPIC array which measures methylation of over 850,000 CpG sites^58,59^) on combination ATRA and ATO treated NB4 cells and patient cells. This would reveal if the combination treatment induces sustained gene expression and methylation changes of other target genes with altered expression, while inducing limited genome-wide methylation changes (as suggested by the surrogate *LINE-1* methylation levels measured here). Thus far, genomic analyses have been primarily focused on the effects of ATRA only in APL cells^15,16^; however, with the increasing utilization of ATRA and ATO in the clinic, the effects of the combination of ATRA and ATO on the epigenome and transcriptome needs to be understood. Differences in the effects of combination treatment may be crucial for APL patients who are resistant to therapy and experience disease relapse^60^. It is also possible that methylation changes of certain target genes (e.g. *RARβ, TGM2*), could be used as a predictor of complete response. Therefore, an increased understanding of the role of epigenetics in APL treatment response may help in the development of novel strategies to overcome treatment failure, and may also lead to strategies for the application ATRA-based therapies in other cancers^61^. Potential strategies include the use of demethylating agents such as decitabine or histone decacetylase inhibitors such as vorinostat^62^, which may improve on the demethylating effects demonstrated in our study.

## Supplementary information

Supplemental methods, figures and tables
