# Supplementary material for: Retinoic acid and arsenic trioxide induce lasting differentiation and demethylation of target genes in APL cells
Source: Sci Rep. 2019 Jul 1;9:9414. doi: 10.1038/s41598-019-45982-7 (PMC6602962; doi:10.1038/s41598-019-45982-7)
Supplement: Supplementary file 1 — Supplemental methods, figures and tables [file 41598_2019_45982_MOESM1_ESM.pdf]

SUPPLEMENTARY INFORMATION: SUPPLEMENTARY METHODS, FIGURES, AND TABLES

Retinoic acid and arsenic trioxide induce lasting differentiation and demethylation of target genes in APL cells

Thomas T Huynh[1#], Mohammad Sultan[1#], Dejan Vidovic[1], Cheryl A Dean[1], Brianne M Cruickshank[1], Kristen Lee[2], Chao-Yu Loung[3], Ryan W Holloway[1], David W Hoskin[1,3], David M Waisman[1,4], Ian C G Weaver[2,3,5,6*], and Paola Marcato[1,3*]

Supplemental Materials and Methods:

Chromatin immunoprecipitation-quantitative PCR (ChIP-qPCR)

At 72h and 96h post treatment termination timepoints, treated NB4 cells were fixed with 1% formaldehyde and the resulting crosslinked protein–DNA complexes were sonicated using a Q800R2 sonicator (QSonica) into 150-250 bp length fragments confirmed using a QIAxcel Advanced System Bioanalyzer. Immunoprecipitation was then performed using rabbit IgG (Diagenode, C15410206) and ChIP-grade rabbit polyclonal IgG antibodies from Diagenode against human H3K9/14ac, H3K9me3 and H3K27me3 (C15410200-10, C15410193-10, C15410195-10). Post immunoprecipitation, the precipitated DNA-protein complexes were dissociated from the protein A conjugated Dynabeads (Invitrogen, Thermo Fisher Scientific) and isolated using a Purelink PCR purification kit (Invitrogen, Thermo Fisher Scientific) along with their respective total input controls (pre-immunoprecipitation DNA). QPCR analysis was then performed using primers targeting the promoter regions of *RARβ* and *TGM2* (Supplemental Table 1) with equipment and reagents described above. Results are expressed as the fold change of the enrichment of the DNA detected under the treatment conditions against the DNA detected under the no treatment conditions. This was determined by dividing the signals obtained from the ChIP by the signals obtained from the total input control sample and normalizing for the DNA detected by the non-immune IgG (negative control).

Supplemental Figures and Tables:

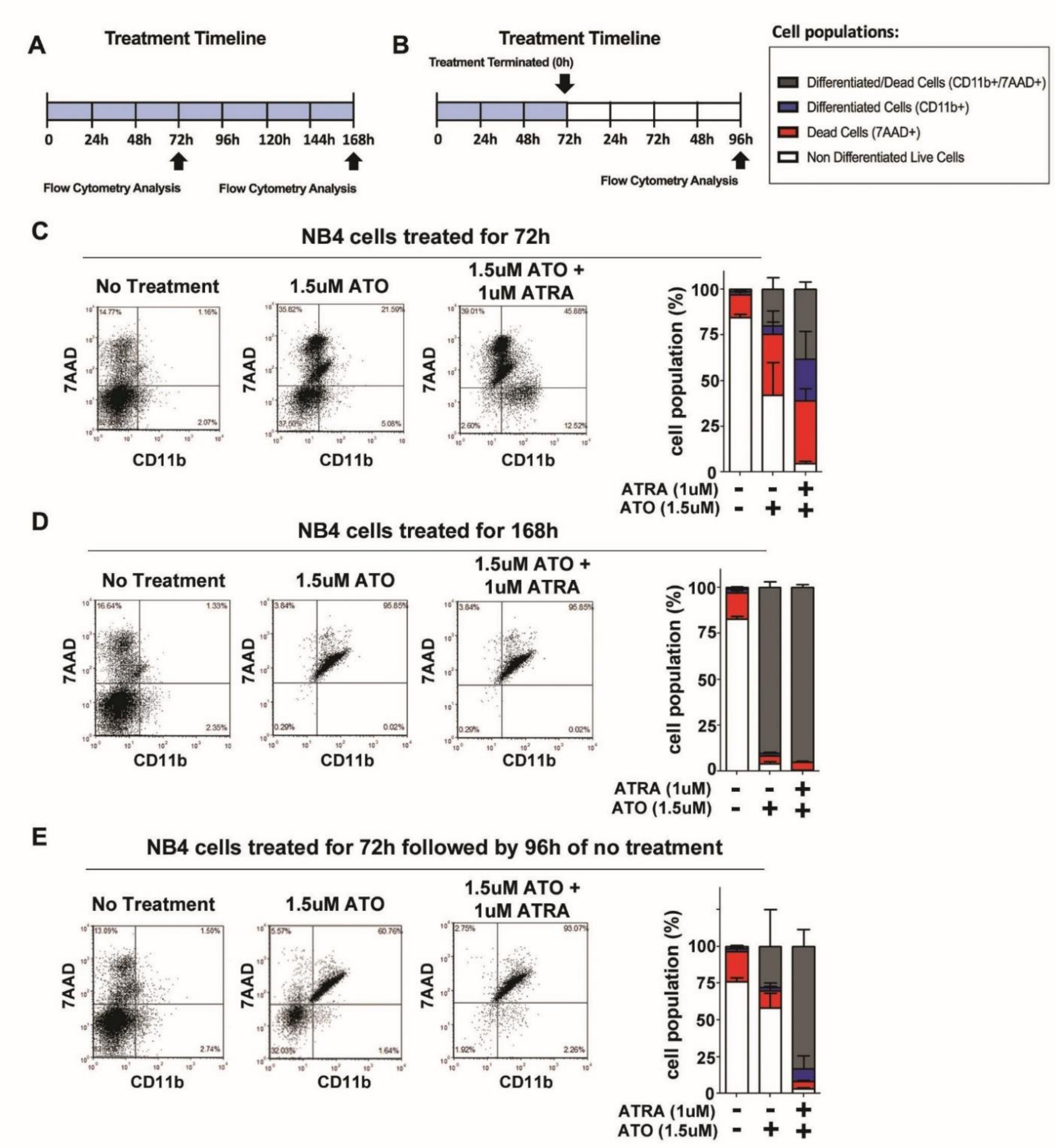

Supplemental Figure 1. 1.5µM ATO treatment induces predominately cell death of NB4 cells, which is mostly sustained 96h post treatment termination and amplified when combined with 1µM ATRA. (A) Schematic of treatment timeline and the timepoints NB4 cells samples were analyzed in C and D. **(B)** Schematic of treatment timeline and the timepoint NB4 cells samples were analyzed in E. **(C, D** and **E)** Representative flow cytometry dot plots of CD11b+, 7-AAD+, and CD11b+/7-AAD+ NB4 cells under no treatment, 1.5µM ATO, 1.5µM ATO + 1µM ATRA treatment after 72h of continuous treatment (**C**), or 168h of continuous treatment (**D**), or after 72h treatment and subsequent 96h post treatment termination. **(C, D** and **E)** The stacked bar graphs summarize the results of dot plots (n=4, error bars represent standard deviation).

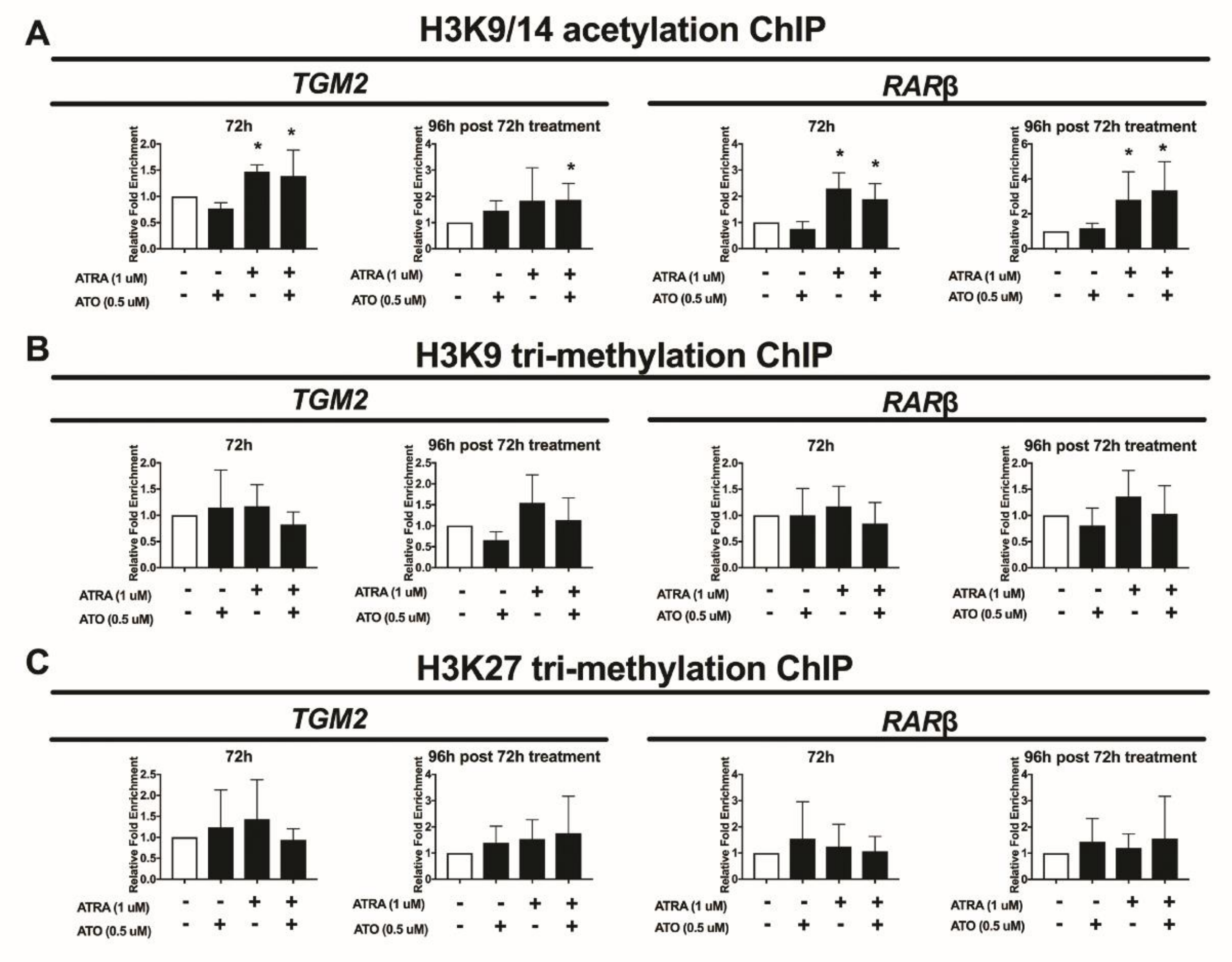

Supplemental Figure 2. ATRA induces sustained enrichment of H3K9/14ac at *TGM2* and *RARβ* promoters in NB4 cells. H3K9/14ac **(A)**, H3K9me3 **(B)**, and H3K27me3 **(C)** enrichment at *TGM2* and *RARβ* promoters as measured by QPCR following ChIP with antibodies specific to the histone modification in NB4 cells following 72h of 0.5µM ATO, 1µM ATRA, or the combination treatment and subsequent 96h post treatment termination. Error bars represent standard deviation, significance determined using one-way ANOVA with multiple comparisons, p value < 0.05 indicated by *, n=4.

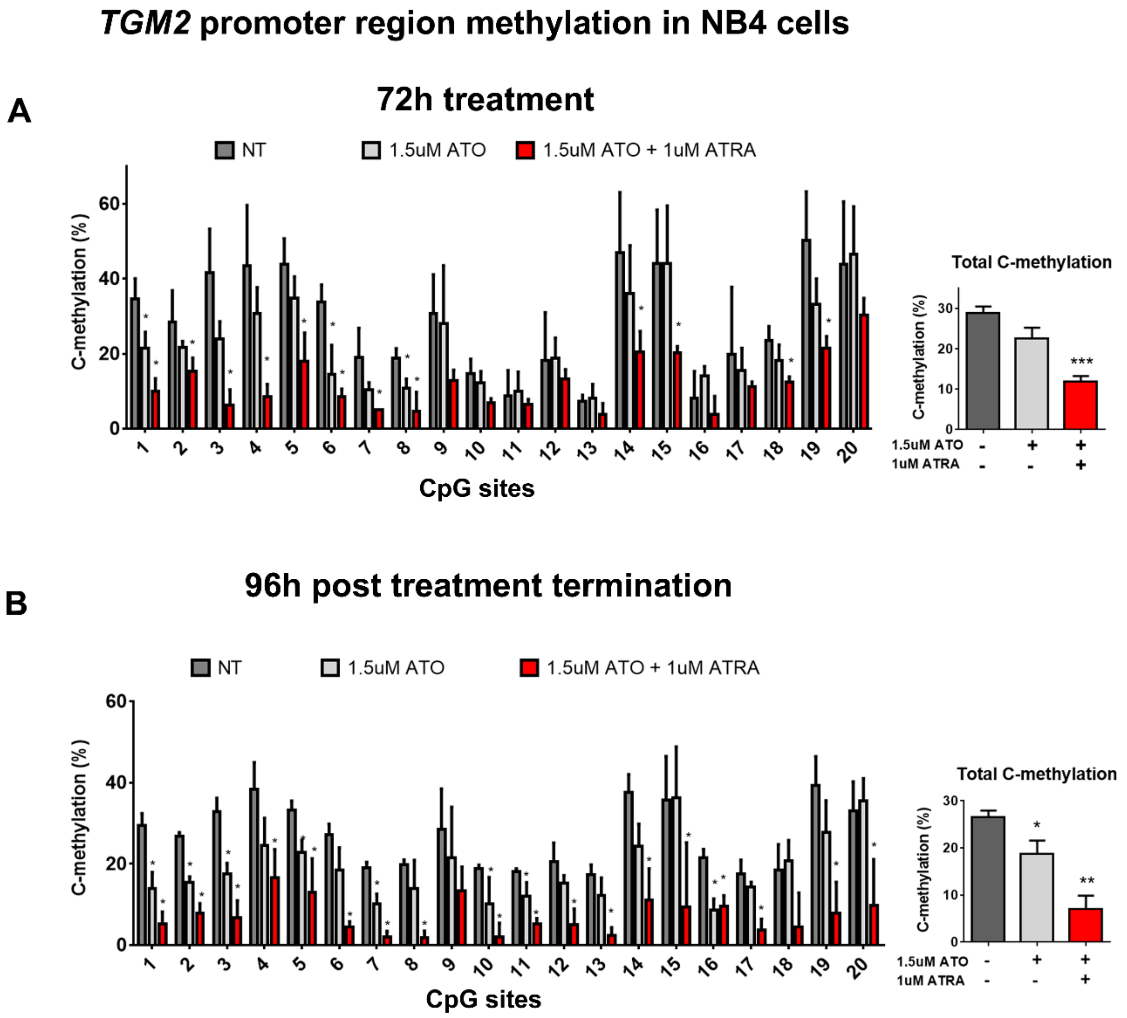

Supplemental Figure 3. 1.5µM ATO reduces DNA methylation of the CpG island in the promoter region of *TGM2* in NB4 cells to a greater degree when combined with 1µM ATRA. (A and B) The methylation percentage of the individual 20 CpG sites and total C-methylation percentage of the region in NB4 cells following 72h of treatment **(A)** and subsequent 96h post treatment termination **(B)**. Error bars represent standard deviation, significance determined using one-way ANOVA with multiple comparisons, n=4.

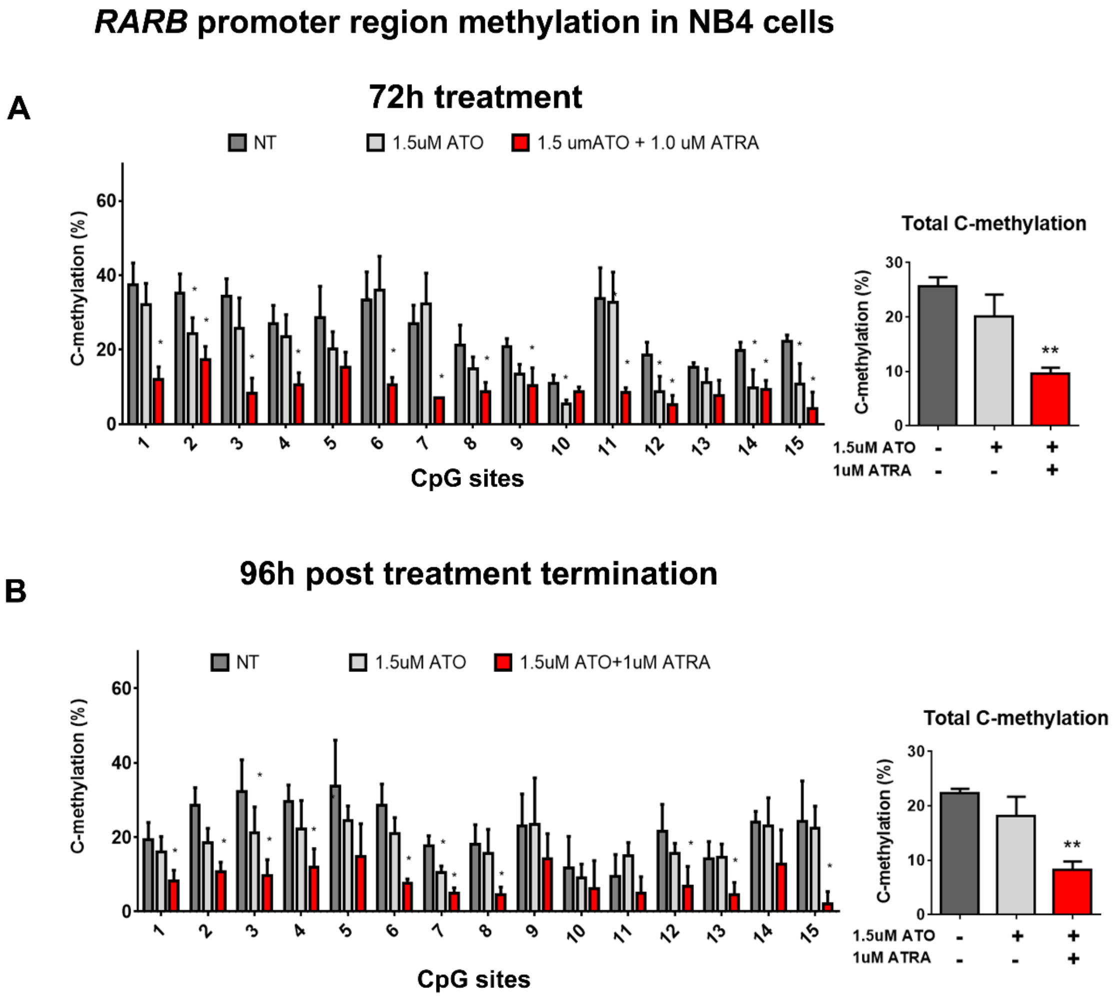

Supplemental Figure 4. 1.5μM ATO reduces DNA methylation of the CpG island in the promoter region of *RARβ* in NB4 cells to a greater degree when combined with 1μM ATRA. (A and B) The methylation percentage of the individual 15 CpG sites and total C-methylation percentage of the region in NB4 cells following 72h of treatment **(A)** and subsequent 96h post treatment termination **(B)**. Error bars represent standard deviation, significance determined using one-way ANOVA with multiple comparisons, n=4.

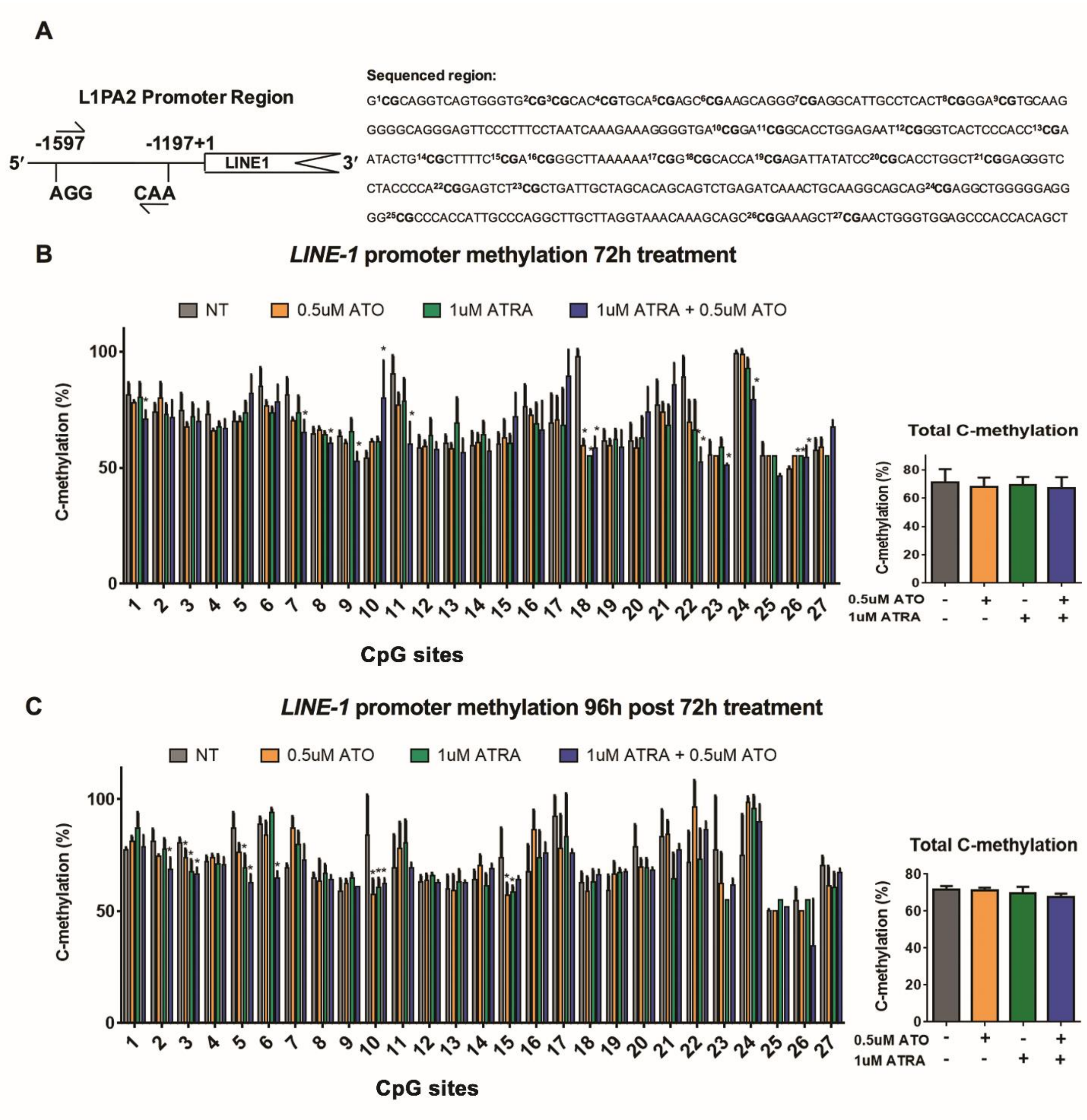

Supplemental Figure 5. Global DNA methylation levels, represented by bisulfite pyrosequencing of *LINE-1*, are unchanged in 1µM ATRA, 0.5µM ATO or combination treatmenat in NB4 cells. (A) Schematic representation of the *LINE-1* subfamily *PA2* and the 27 CpG sites located within the region that were bisulfite pyrosequenced. **(B and C)** The methylation percentage of the individual 27 CpG sites and total C-methylation percentage of the region in NB4 cells following 72h of treatment **(B)** and subsequent 96h post treatment termination **(C)**. Error bars represent standard deviation, significance determined using one-way ANOVA with multiple comparisons, p value < 0.05 indicated by *, n=5.

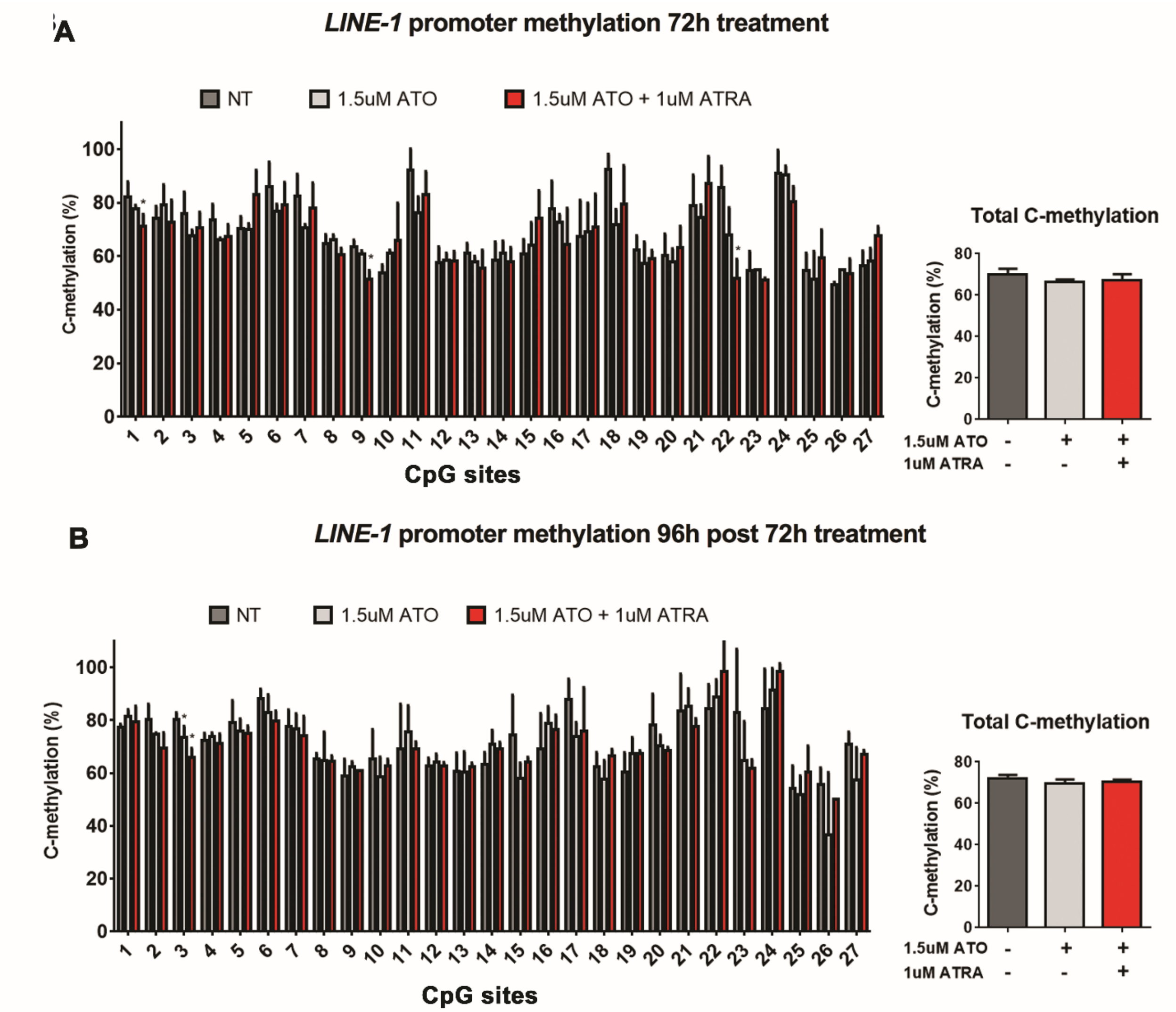

Supplemental Figure 6. 1.5μM ATO (with or without ATRA) does not reduce global DNA methylation levels, represented by bisulfite pyrosequencing of *LINE-1*, in NB4 cells. (A and B) The methylation percentage of the individual 27 CpG sites and total C-methylation percentage of the region in NB4 cells following 72h of treatment **(A)** and subsequent 96h post treatment termination **(B)**. Error bars represent standard deviation, significance determined using one-way ANOVA with multiple comparisons, n=4.

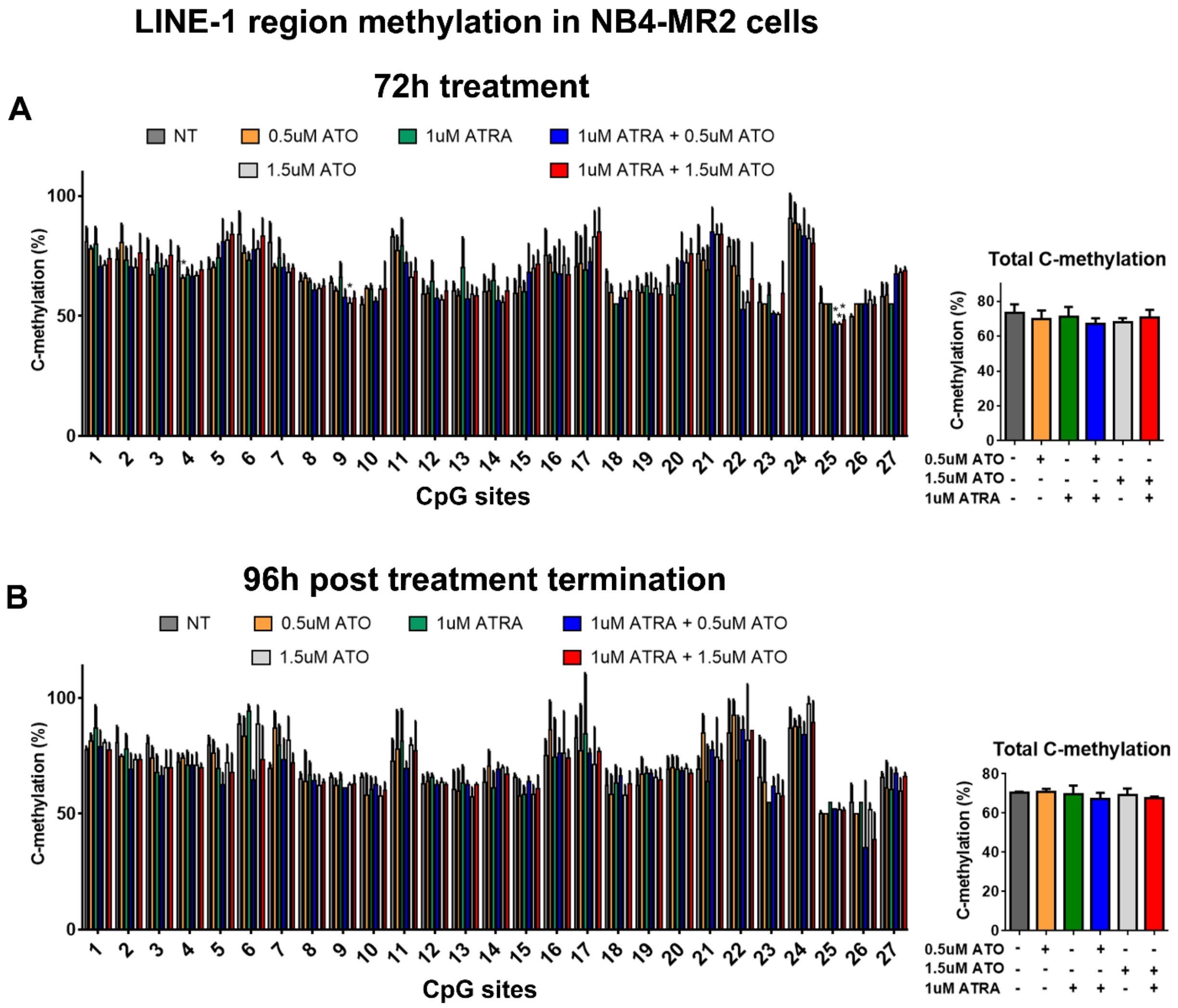

Supplemental Figure 7. Global DNA methylation levels, represented by bisulfite pyrosequencing of *LINE-1*, are unchanged in ATRA, ATO or combination treated NB4-MR2 cells. (A and B) The methylation percentage of the individual 27 CpG sites and total C-methylation percentage of the region in NB4-MR2 cells following 72h of treatment (**A,** n=4) and subsequent 96h post treatment termination (**B**, n=3). Error bars represent standard deviation, significance determined using one-way ANOVA with multiple comparisons.

Supplemental Table 1. Primer sequences for qPCR and ChIP-qPCR analysis

qPCR Primers		
Gene	Forward Primer (5’→3’)	Reverse Primer (3’→5’)
TGM2	GAGCGAAGGGACGTACTGC	GACAAAGGGCGCATCGTACTT
RARβ	TTCTCAGACGGCCTTACCCT	GCTGGTTGGCAAAGGTGAAC
CCL2	GAAAGTCTCTGCCGCCCTT	GGGGCATTGATTGCATCTGG
ASB2	GAGCCGGACATCTCCAACAA	CACCAGAATCTTCACGGCCT
IER3	CAGAGGACGCCCCTAACG	TGTTGCTGGAGGAAAGTGCT
PRTN3	TGCCGGCCACATAACATTTG	CCCCAGATCACGAAGGAGTC
RPL7a	CAAAAGAGACCTCACCCGCT	CAAAAGAGACCTCACCCGCT
RARα	GTGTCACCGGGACAAGAACT	CGTCAGCGTGTAGCTCTCAG
RAB33A	GCTGGTTGGCAAAGGTGAAC	GCTGGTTGGCAAAGGTGAAC
NDUFB10	CTACTACCACCGGCAGTACC	TCTTCCACTGCATTTCGGCT
NCL	GCTGGTTGGCAAAGGTGAAC	GCTGGTTGGCAAAGGTGAAC
MPO	CGCCAACGTCTTCACCAATG	CATGGGCTGGTACCGATTGT
HIST1H2BK	ACCTCCAGGGAGATCCAGAC	TGTACTTGGTGACGGCCTTG
TBP	GGCAACCACTCCACTGTATCC	GCTGCGGTACAAATCCCAGAA
HPRT1	GACCAGTCAACAGGGGACAT	CCTGACCAAGGAAAGCAAAG
ChIP-qPCR Primers		
Gene	Forward Primer (5’-3’)	Reverse Primer (3’-5’)
TGM2	CTTCACCGAGCCTCAGTTTC	GATAAGCCCCAGAGGTCACA
RARβ	GGGAGAGAAGTTGGTGCTCAA	CACAAGCCGGCGTTTTCTTT

Supplemental Table 2. Primers for bisulfite pyro-sequencing

Gene	Forward Primer (5’→3’)	Reverse Primer (3’→5’)	Sequencing Primers
TGM2	TTGGTGTTTTTTTTTTT TGTTGATGAG	(Biotin)TCTCCTCCTCCC TAAACAAAAT	TTGTTGATGAGG TGG
RARβ	GTTAAAGGGGGGATT AGAATTT	(Biotin)AACTCTACCCC TTTTTTAACA	GGGGGATTAGA ATTTTTTTAT
LINE-1	AGGGAGAGTTAGATA GTG	(Biotin)AACTATAATAA ACTCCACCC	GGAGAGTTAGAT AGTGG
